# Recreational 3 × 3 basketball elicits higher heart rate, enjoyment, and physical activity intensities but lower blood lactate and perceived exertion compared to HIIT in active young adults

**DOI:** 10.5114/biolsport.2023.122478

**Published:** 2023-02-01

**Authors:** Daniele Conte, Inga Lukonaitiene, Kestutis Matulaitis, Audrius Snieckus, Audinga Kniubaite, Rasa Kreivyte, Sigitas Kamandulis

**Affiliations:** 1 Institute of Sport Science and Innovations, Lithuanian Sports University, Kaunas, Lithuania; 2 Department of Coaching Science, Lithuanian Sports University, Kaunas, Lithuania

**Keywords:** Cardiovascular fitness, Creatine kinase, Health, Physical activity, Training adherence

## Abstract

This study aimed to assess and compare the physiological [percentage of maximal heart rate (%HR_max_), blood lactate (BLa), creatine kinase (CK)], hormonal (testosterone, cortisol), psychological [rating of perceived exertion (RPE), enjoyment] and physical [percentage of moderate-to-vigorous physical activity (%MVPA) and vigorous activity (%VA)] responses of recreational 3 × 3 basketball (3 × 3BB) and high-intensity interval training (HIIT) in active young adults. Twelve apparently healthy male recreational basketball players (age: 23 ± 3 years; body mass: 82 ± 15 kg; stature: 188 ± 15 cm) completed a 3 × 3BB match and HIIT with similar duration. %HR_max_, %MVPA and %VA were monitored during the protocols, while BLa, cortisol, and testosterone were measured before and after each protocol. CK was measured before the protocols and at 24 h, while RPE and enjoyment were assessed at the end of each protocol. 3 × 3BB elicited higher %HR_max_ (p < 0.001; d = -1.6, large), %MVPA (p < 0.001; d = 2.7, very large), %VA (p = 0.030; d = 0.8, moderate), enjoyment (p = 0.014; r-value = -0.500, large), and lower RPE (p = 0.024; r-value = -0.462, moderate) compared to the HIIT condition. Moreover, higher values of BLa were found in HIIT compared to 3 × 3BB at post-condition (p = 0.020; r-value = -0.601, large), while CK analysis showed only an increase within the HIIT condition (p = 0.020; r-value = -0.599, large). A time effect was found for both testosterone (p < 0.001, η^2^_p_ = 0.526, moderate) and cortisol (p = 0.005, η^2^_p_ = 0.743, strong), while no between-condition effect or interaction was found (p > 0.05). 3 × 3BB elicits higher %HR_max_, enjoyment, and physical activity intensities but lower BLa and RPE compared to HIIT in active young adults and might be considered as a potentially suitable activity to increase participants’ health status.

## INTRODUCTION

Possessing adequate physical activity levels is fundamental for young adults for the prevention of chronic diseases, which are among the most important causes of mortality worldwide [1]. However, more than 50% of young adults fail to meet the international physical activity guidelines [2]. Therefore, one of the main goals for sport and health scientists is to design and select suitable physical activities for young adults that could encourage their participation, and in turn produce positive effects on their health status.

In the last decade, the beneficial effects on fitness outcomes of short training methods such as high-intensity interval training (HIIT) have been widely studied [3, 4]. Typical HIIT sessions last ~10 min with a work-to-rest ratio ~1:1 (e.g. 30 s work and 30 s rest) and can stimulate similar or higher physiological adaptations compared to traditional aerobic training with longer duration such as running or cycling-based activities [4]. Moreover, HIIT programmes can be characterized by several activities including endurance, resistance training, sport, combat and brain activities in the attempt to be more appealing for young adults [5]. Overall, the use of these HIIT typologies was positively rated by young adults such as university students possibly due to their short duration and the selection of the activities that motivate their participation [5]. Consequently, HIIT activities have been suggested as one of the best methodologies to increase participants’ adherence to training programmes and in turn increase their physical activity levels.

An alternative way to increase the physical activity level among young adults is the practice of team sports such as football, hand-ball, or basketball [6]. Specifically, playing football small-sided games has shown a higher potential to involve and motivate sedentary people to engage in physical activity compared to other conventional training (e.g. running or cycling) [7]. Considering basketball, a previous investigation revealed that 3 months of basketball small-sided games (3v3) played on half and full court improved the overall fitness profile in untrained adult men (20–42 years) [8]. However, it should be considered that the training sessions lasted ~ 75 min with basketball small-sided games comprising 4 × 12 min games interspersed by 3-min breaks [8]. Potentially, the implementation of team sports activities with shorter durations like those implemented during HIIT modalities can be more appealing for individuals willing to be involved in regular physical activity. In fact, activities with long duration might decrease the adherence in regular physical activity programmes of young adults [9] since it has been shown that the perceived lack of time is one of the barriers for participation in regular exercise activities [10]. Therefore, minimizing the time dedicated to training although maintaining the physical and physiological response potentially leading to positive adaptations might be essential to increase the physical activity levels in young adults.

In this regard, recreational 3 × 3 basketball matches could be considered as a valuable tool to elicit adequate physiological responses and increase young adults’ involvement in physical activities since they possess the advantages of being a regular team sport (e.g. ball involvement and teammate interactions) and having a short duration similar to typical HIIT typologies (10 min of live time). To the best of our knowledge, no previous study assessed whether recreational 3 × 3 basketball could produce similar physical and physiological responses to those elicited by HIIT modalities in young adults. This information seems essential since it might clarify whether 3 × 3 basketball can be considered as a valuable team sport activity to potentially elicit positive health-related adaptations. Therefore, the aim of this study is to compare the physiological, hormonal, psychological and physical responses of recreational 3 × 3 basketball and HIIT in young adults.

## MATERIALS AND METHODS

### Participants

Twelve apparently healthy, male, recreational basketball players (age: 23 ± 3 years; body mass: 82 ± 15 kg; stature: 188 ± 15 cm; %fat mass: 10.6 ± 6.3) were recruited to participate in this study. An apriori analysis indicated that the present study is sufficiently powered using α = 0.05, β = 0.80 and an effect size = 1.0 (G*Power, version 3.1.9.2; University of Dusseldorf; Germany) based on previous research investigating differences in enjoyment levels between HIIT and moderate intensity continuous exercise [11]. Participants completed the Physical Activity Readiness Questionnaire (PAR-Q) prior to participation to rule out contraindications to participation and a custom-made questionnaire in which they reported weekly physical activity levels in line with the recommendations provided by the World Health Organization [12]. Overall, the selected participants can be classified as “habitually active, physically fit and recreationally trained” based on the classification provided by Russel et al. [13]. The procedures, benefits, and risks involved in participation were explained to each player before participation and informed written consent was obtained from all players. The procedures received approval from the ethics committee of the Lithuanian Sports University [approval number: BNL-TRS(B)-2022–449].

### Design

A descriptive within-subject design encompassing five experimental sessions was used to assess the acute physiological, hormonal, psychological, and physical responses in recreational 3 × 3 basketball and HIIT modalities. In the first session, participants’ anthropometric characteristics (stature, body mass and %fat mass) and maximal heart rate (HR_max_) were assessed. Furthermore, players were required to complete 4 min of both recreational 3 × 3 basketball and HIIT activities to familiarize themselves with the study procedures after receiving detailed explanations by the research staff members. Participants also familiarized themselves with all other procedures such as saliva and blood collections, and with the adopted scales.

**FIG. 1:**
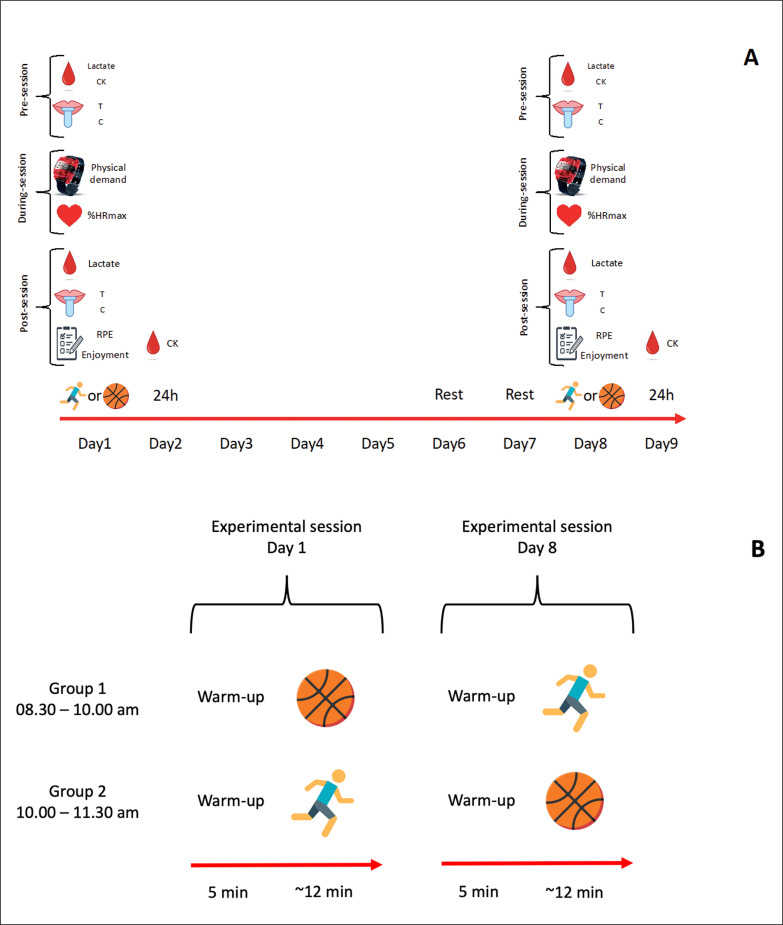
**A** indicates the full study design. **B** indicates the organization in the experimental sessions on day 1 and day 8. Note: 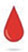 = blood collection; CK = creatine kinase; 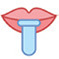 = saliva collection; T = testosterone; C = cortisol; 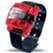 = monitoring physical activity intensities; 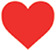 = monitoring heart rate; %HR_max_ = percentage of maximal heart rate; 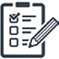 = assessment of psychological demand; RPE = rating of perceived exertion; 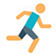 = high-intensity interval training; 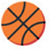 = recreational 3 × 3 basketball.

Following familiarization, four experimental sessions were conducted over 9 days with participants divided into two groups (Figure 1). On day 1 and day 8, participants were randomly and equally divided into two groups performing either recreational 3 × 3 basketball or HIIT, while on day 2 and day 9 participants’ conditions at 24 h were assessed (Figure 1). All experimental sessions were scheduled at the same time of the day (group 1: 8.30 am – 10.00 am; group 2: 10.00 am – 11.30 pm) to avoid issues due to circadian rhythms. Participants were also instructed not to perform any physical activity in the 48 h preceding the recreational 3 × 3 basketball or HIIT protocols and in the following 24 h, to maintain regular sleeping patterns and diet, and to avoid caffeine and alcohol prior to each experimental day. Moreover, before each experimental session, participants completed a questionnaire assessing their compliance with pre-test instructions.

### Procedures

#### Recreational 3 × 3 basketball and HIIT protocols

The recreational 3 × 3 basketball matches were played following the official rules of 3 × 3 basketball (https://fiba3×3.com/en/rules.html) with a 10-min duration of live time (or match over when one of the two teams reached 21 points). The studied matches had an average duration of 10.2 min. Players were notified about the score, and a 12-s shot clock was used as in official competitions. It was played on the half court of an indoor regular-sized basketball court with a wooden floor and with the official ball for international 3 × 3 basketball competitions (size 6). Teams were generated by the research staff based on players’ positions with no tactical indications provided. No substitutions were allowed during the match since teams were composed of 3 players and not 4 players as in official competitions.

The Gym HIIT was selected for the purpose of this study as the HIIT typology, since it has previously demonstrated its efficacy and feasibility of use in the university setting, with students rating this HIIT typology the most favourably [5]. Briefly, the Gym HIIT included a 12-min duration and a work-to-rest ratio of 30 s:30 s with a combination of various activities (i.e. push-ups, shuttle sprints, squat jumps, shuttle side-step, sit-ups and jumping jacks) (Table 1). Within the 30-s bout, participants were required to complete as many rounds of activities as possible (for instance, in the first bout they would repeat as many times as possible the 4 push-ups +10 m shuttle sprints). Both recreational 3 × 3 basketball and HIIT protocols were preceded by a 10-min standardized warm-up consisting of jogging and a series of mobility exercises and dynamic stretching followed by basketball-specific drills including dribbling and shooting.

**TABLE 1:** Gym HIIT sequences and activities (total time 12 min).

Activities	Time	Sets
4 × Push-ups + 10 m Shuttle Sprint	30 s	4

Rest	30 s	

4 × Squat Jump + 10 m Shuttle Side-step	30 s	

Rest	30 s	

4 × Sit-Ups + 10 × Jumping Jacks	30 s	

Rest	30 s	

#### Physiological and hormonal responses

Participants’ HR_max_ was assessed in the first experimental session via the 30–15 Intermittent Fitness test specifically developed for basketball, [13] which has been largely used to assess basketball players’ HR_max_ [14–16]. Briefly, the test consisted of 30-s shuttle runs performed across the regular-sized basketball court, interspersed by 15 s passive recovery periods. Players were asked to run back and forth on the court completing as many stages as possible following a pre-recorded beep with incremental speed. The test ended when players could no longer maintain the required speed and the heart rate registered at the final stage was considered as HR_max_. During both recreational 3 × 3 basketball and HIIT, the %HR_max_ was measured using H10 Bluetooth heart-rate strap (Polar Electro Oy, Kempele, Finland) sampling at 1 s, which is considered one of the gold standards in assessing HR [17]. Each strap was connected via Bluetooth to each participant’s smartphone using the app Polar Beat, which has been previously used [17]. At the end of each activity, data were transferred onto researchers’ Polar cloud account, and successively downloaded on Excel spreadsheets for further analysis.

Moreover, blood lactate was measured as an objective internal response. Ear lobe blood samples were taken before (in resting condition) and 1 min and 5 min after the completion of each protocol, with the highest value indicated as the peak and used for further analysis. Blood samples were analysed using a Lactate Pro 2 CT-1730 analyser (Arkray Inc., Kyoto, Japan) [18].

Creatine kinase (CK) was measured to assess the muscle damage before and 24 h after each experimental session at the laboratories of the Lithuanian Sports University. About 5 mL of blood was drawn from the median cubital vein, with samples centrifuged immediately after, and analysed using a Spotchem EZ SP-4430 biochemical analyser (Menarini Diagnostics, Winnersh, Wokingham, UK) using soft reagent strips (Arkray Factory, Inc., Shiga, Japan). Plasma enzyme activity was reported as international units per litre (IU × L^-1^). The normal reference range for human plasma CK using this method is 56–244 IU × L^-1^ according to the manual provided with the analyser.

Saliva samples were used to measure testosterone and cortisol levels collected before and after recreational 3 × 3 basketball and HIIT protocols. Participants were instructed to not eat, brush their teeth, or consume any drink other than still water in the 90 min prior to saliva collection. Before saliva collection, each participant rinsed his mouth with distilled water, waited in a seated position for 30 s, and then removed all saliva from his mouth. Afterwards, they waited for ~15 min before the saliva specimen was collected into 15-mL ultrapure polypropylene SaliCap tubes through polypropylene straws (IBL International, Hamburg, Germany). Saliva samples was then stored at -20°C before determining testosterone and cortisol levels via an enzyme-linked immunoassay (IBL International, Hamburg, Germany) following the manufacturer’s instructions. Samples were analysed in duplicate with an intra-assay coefficient of variation of 1.34% and 1.92% for testosterone and cortisol, respectively.

#### Perceptual and psychological responses

The rating of perceived exertion (RPE) was measured for each participant at the end of each protocol using the Category Ratio (0–10) RPE scale [19]. Furthermore, participants’ enjoyment was assessed using the Exercise Enjoyment Scale (EES) [20]. Both scales were previously adopted with this population involved in activities with similar typology and intensity [9].

#### Physical responses

During each activity, participants’ physical activity levels were assessed using 30 Hz accelerometers (GT3X model, Actigraphs, Pensacola, Florida, USA) attached to the right hip of the participants underneath clothing and secured with an elastic band. Using proprietary software (Actilife, version 6.13.4 for Windows), the time in minute spent at each physical activity intensity was calculated based on recommended vector magnitude cut points [21] and expressed in percentages. Specifically, the percentages of time spent in moderate-to-vigorous physical activity (MVPA) and vigorous activity intensities were determined similarly to previous studies in recreational team sports [22].

#### Statistical analysis

Mean and standard deviation (SD), median and inter-quartile range (IQR) and percentages (%) were calculated as descriptive statistics. Data normality for each continuous dependent variable was assessed using the Shapiro-Wilk test, which revealed non-normally distributed data for blood lactate and CK. Therefore, a paired-sample t-test was used to assess the differences between recreational 3 × 3 basketball and HIIT conditions in %HR_max_, %MVPA and %vigorous activity, while a 2 × 2 (time × condition) repeated measure ANOVA was used for testosterone and cortisol values. A non-parametric approach was used for the non-normally distributed variables and ordinal variables (i.e. RPE and enjoyment). Specifically, the Wilcoxon signed-rank test was used to assess the differences in RPE and enjoyment between recreational 3 × 3 basketball and HIIT conditions. Moreover, the Wilcoxon signed-rank test with Bonferroni correction was used to assess the between- and within-condition differences for blood lactate and CK. Cohen’s d was used as an effect size measure for the paired t-test and values were interpreted according to Hopkins’ benchmarks as follow: trivial ≤ 0.20; small = 0.2–0.59; moderate = 0.60–1.19; large = 1.20–1.99; very large ≥ 2.0 [23]. Moreover, partial eta-squared (η^2^_p_) was used as a measure of effect size for the 2 × 2 repeated measure ANOVA and values were interpreted as no effect (η^2^_p_ < 0.04), a minimum effect (0.04 < η^2^_p_ < 0.25), a moderate effect (0.25 < η^2^_p_ < 0.64) and a strong effect (η^2^_p_ > 0.64) [24]. Finally, the r-value [Z/SQRT(N)] was adopted as an effect size measure for non-parametric statistics and interpreted according to Cohen’s benchmarks considering 0.1, 0.3 and 0.5 as a small, medium and large effect size, respectively [25]. The α level was set at 0.05 and statistical analyses were performed using the software IBM SPSS for Windows (version: 28.0.1.0, IBM. Corp; Armonk, NY) and Jamovi for Windows (version: 2.3.5; Sydney, Australia; retrieved from https://www.jamovi.org).

## RESULTS

### Physiological and hormonal responses

**FIG. 2:**
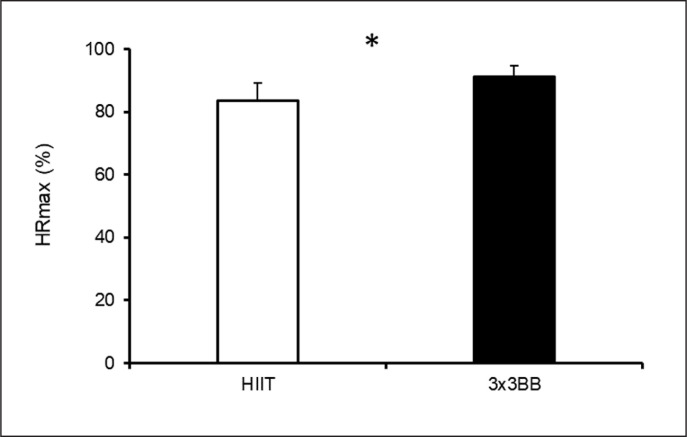
Percentage of maximal heart rate (%HR_max_) during high-intensity interval training (HIIT) and recreational 3 × 3 basketball (3 × 3BB). Note: * = indicates p < 0.001.

**FIG. 3:**
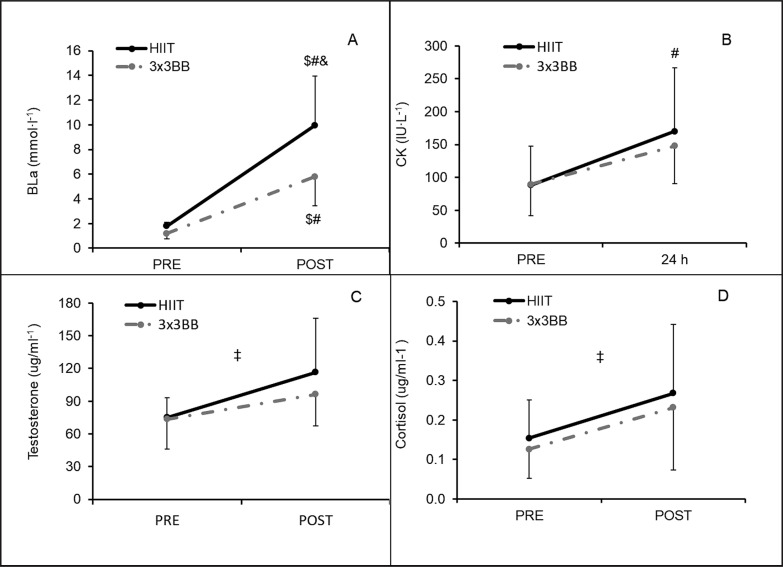
Difference between high-intensity interval training (HIIT) and recreational 3 × 3 basketball (3 × 3BB) at different time points for: **A** – blood lactate (BLa); **B –** creatine kinase (CK); **C –** testosterone; **D –** cortisol. Note: non-parametric statistics was applied calculating median ± interquartile range (IQR) for BLa (HIIT-pre = 1.8 ± 0.2; HIIT-post = 10.0 ± 6.4; 3 × 3BB-pre = 1.2 ± 0.4; 3 × 3BB-post = 5.8 ± 3.1) and CK (HIIT-pre = 81.3 ± 32.3; HIIT-24h = 166.5 ± 96.6; 3 × 3BB-pre = 70.2 ± 60.2; 3 × 3BB-24h = 147.0 ± 71.7); # = indicates difference compared to HIIT-pre (p < 0.05); $ = indicates difference compared to 3 × 3BB-pre (p < 0.05); & = indicates difference compared to 3 × 3BB-post (p = 0.020); ‡ = indicates time effect for testosterone (p < 0.001) and cortisol (p = 0.005).

The recreational 3 × 3 basketball condition elicited a higher %HR_max_ compared to HIIT (p < 0.001; mean difference (95%CI) = -7.6 (-10.7; -4.5); Cohen’s d (95%CI) = -1.6 (-2.4; -0.7) – large) (Figure 2). Moreover, lactate analysis showed a significant difference across all the between and within comparisons (p < 0.05; r-values: large) except for the comparison between values before HIIT and recreational 3 × 3 basketball (p = 0.222; r-value: medium) (Figure 3). Furthermore, the CK analysis showed only a significant difference within the HIIT condition between pre- and 24 h time-points (p = 0.020; r-value = -0.599, large) (Figure 3). A time effect was found for both testosterone (p < 0.001, η^2^_p_ = 0.526, moderate effect) and cortisol (p = 0.005, η^2^_p_ = 0.743, strong effect), while no effect was found comparing the two conditions and their interaction (p > 0.05) (Figure 3).

### Perceptual and psychological responses

**FIG. 4:**
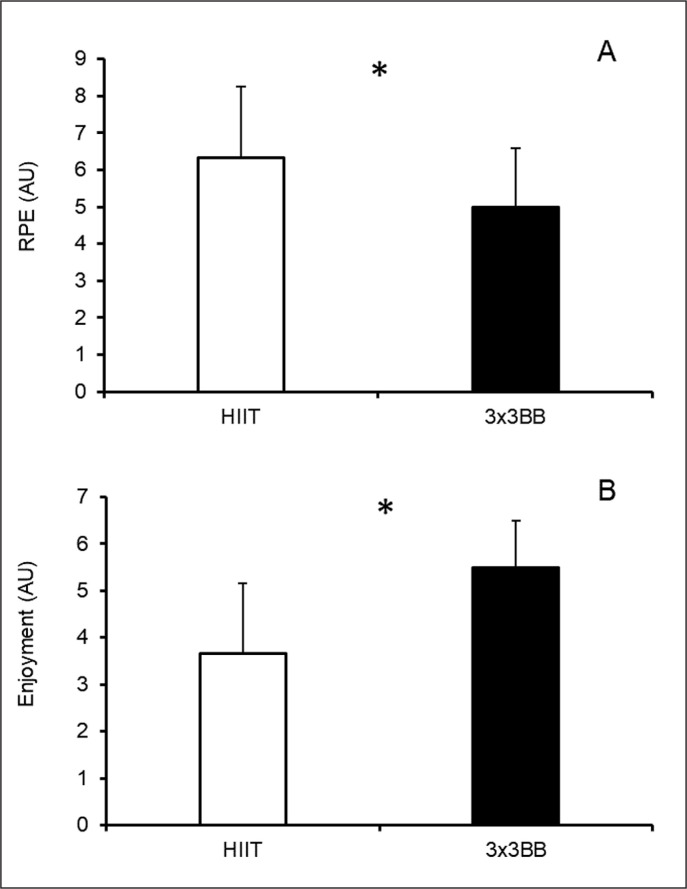
Difference between high-intensity interval training (HIIT) and recreational 3 × 3 basketball (3 × 3BB) in: **A** – rating of perceived exertion (RPE); and **B** – enjoyment. Note: non-parametric statistics was applied calculating median ± interquartile range (IQR) for both RPE (HIIT = 6.5 ± 2.3; 3 × 3BB = 5.0 ± 1.5) and enjoyment (HIIT = 4.0 ± 3.0; 3 × 3BB = 6.0 ± 1.0); * = indicates p < 0.05.

Players perceived a higher RPE in the HIIT condition compared to the recreational 3 × 3 basketball condition (p = 0.024; r-value = -0.462, medium) (Figure 4). Conversely, players reported higher enjoyment for the recreational 3 × 3 basketball condition compared to the HIIT condition (p = 0.014; r-value = -0.500, large) (Figure 4).

### Physical demand

Playing recreational 3 × 3 basketball elicited a higher physical activity intensity with very large and moderate differences in %MVPA (p < 0.001) and %vigorous activity (p = 0.030), respectively (Table 2).

**TABLE 2:** Differences in the physical activity intensities between recreational 3 × 3 basketball and HIIT

Physical activity intensities	Activities			Activity Comparison (3 × 3BB vs. HIIT)		

	HIIT	3 × 3BB	p	Mean difference (95% CI)	ES (95%CI)	Interpretation
MVPA (%)	75.6 ± 6.2	95.6 ± 1.9	< 0.001	20.0 (15.1; 24.9)	2.7 (1.4; 4.1)	Very Large
Vigorous activity (%)	61.4 ± 3.0	71.9 ± 15.2	0.030	10.5 (1.2; 19.9)	0.8 (0.1; 1.4)	Moderate

Data are presented as mean ± SD. Abbreviations: MVPA = moderate-to-vigorous physical activity; HIIT = high-intensity interval training; 3 × 3BB = recreational 3 × 3 basketball; 95% CI = 95% confidence interval; ES = effect size

## DISCUSSION

The aim of this study was to assess whether recreational 3 × 3 basketball could provide physiological, hormonal, psychological, and physical responses comparable to an activity with similar duration recognized to elicit health benefits in young adults such as the Gym HIIT. The main results revealed that recreational 3 × 3 basketball elicited greater heart rate responses, enjoyment, and physical activity intensities but lower lactate, CK and RPE compared to HIIT in young adults. These results suggest that the use of 3 × 3 basketball as recreational activity could potentially produce health benefits in young adults.

### Physiological and hormonal response

The exercise intensities reached during both recreational 3 × 3 basketball and HIIT might be classified as vigorous based on the classification of various organizations such as the American College of Sports Medicine (ACSM) [26], which identified a vigorous exercise intensity > 77%HR_max_ or ranging between 80% and 90% HR_max_ [27, 28]. Our results also showed that Gym HIIT elicited 83.6 ± 5.5% HR_max_, which is in line with the previous investigation of Eather et al. [5] documenting an average of 82.2% HR_max_ during the Gym HIIT and HIIT modalities following the same structure. This intensity has been shown to induce positive adaptation in cardiorespiratory fitness and upper body muscular fitness [5]. Interestingly, recreational 3 × 3 basketball elicited a higher average %HR_max_ (91.2 ± 3.5) compared to the Gym HIIT. A possible reason for this result might be that no substitutions were allowed during the recreational 3 × 3 basketball matches, reducing the number of stoppage times compared to the HIIT activity, which had a fixed work-to-rest ratio of 1:1 (30 s work and 30 s rest). It should be considered that the cardiovascular intensities produced by recreational 3 × 3 basketball were also higher than those indicated during ~75 min of recreational 3 × 3 basketball activity composed of 4 × 12 min bouts with 3 min rest in between and played either on full (83.8 ± 6.0 %HR_max_) or half court (84.5 ± 2.9 %HR_max_), which was also shown to produce beneficial effects on participants’ cardiorespiratory fitness, systolic blood pressure, and glucose tolerance [8]. Overall, these results highlight that health practitioners could consider the use of recreational 3 × 3 basketball as a potential activity able to increase the cardiovascular fitness in young adults similarly or even superiorly than the proposed HIIT modality.

Measuring blood lactate concentration can provide indications about the metabolic stress of training and the contribution of the anaerobic glycolytic pathway [29]. It is hard to make a comparison between the blood lactate concentrations documented in the HIIT typology proposed in this study and the HIIT adopted in previous studies due to different activity typologies and training regimes adopted. Nevertheless, it can be noted that the proposed HIIT typology induced a high metabolic response with average blood lactate concentration of ~10 mmol × L^-1^, which is higher than HIIT including all-out cycling protocols [30] or jumping exercises with little or no rest [31] (~9 mmol × L^-1^) and lower compared to other HIIT typologies including more strenuous activities such as Tabata training [9] or CrossFit sessions [32] (12–15 mmol × L^-1^). In contrast, recreational 3 × 3 basketball elicited a remarkably lower blood lactate concentration of ~6 mmol × L^-1^, which is in line with the values documented in elite male (6.33 ± 2.43 mmol × L^-1^) and female (6.09 ± 2.24 mmol∙L^-1^) 3 × 3 basketball players participating in various European and world championships [33]. Overall, these results highlight the higher metabolic stress imposed by the HIIT activity compared to recreational 3 × 3 basketball. It is hard to define the mechanisms involved in this difference of lactate production, but it can be speculated that strength exercises (although with body weight) involving both upper and lower limbs performed during the HIIT induced higher muscular stress, producing an accumulation of protons and reactive oxygen species and consequently higher acidosis in the exercising muscles [34].

The suggested higher muscular stress during HIIT compared to recreational 3 × 3 basketball is also supported by the statistically significantly higher plasma CK values (although with a small effect) found in this study. To the best of the authors’ knowledge, no previous investigation has assessed the plasma CK values following Gym HIIT and 3 × 3 basketball (both recreational and official competitions), making any possible comparison with previous literature difficult. Nevertheless, our outcomes documented that both activities produced lower CK values measured at 24 h (Gym HIIT: 169.9 ± 96.6 IU × L^-1^ and recreational 3 × 3 basketball: 147.7 ± 57.1 IU × L^-1^) compared to other studies investigating longer activities such as a simulated basketball match (408.6 IU × L^-1^) [35] and muscle damage induced exercises (~800 IU × L^-1^) [36]. Furthermore, the changes in CK from pre- to post-activity measured at 24 h for the HIIT typology adopted in the current study (~80 IU × L^-1^) were lower compared to HIIT adopting the same work-to-rest ratio and durations (30 s:30 s) but ending at voluntary exhaustion (~100 IU × L^-1^) [37]. These outcomes suggest that the short (~12 min) activities proposed in this study did not induce a high level of muscle damage in active young adults, suggesting that limited recovery time might be used between these activities in this population. The slightly higher level of muscle damage found in the Gym HIIT modality might be due to the higher involvement of strength exercises compared to recreational 3 × 3 basketball, involving a higher number of muscle groups, also including the upper limbs (i.e. push-ups). These data should also be confirmed in a sedentary population, which might reach different muscle damage levels compared to the active participants involved in the current study.

To the best of our knowledge, this is the first study comparing the effect of playing recreational 3 × 3 basketball and performing a HIIT session on salivary testosterone and cortisol, which have been considered as sensitive biomarkers to monitor the anabolic and catabolic responses of HIIT [38] and basketball activities [14, 16]. Indeed, assessing the responses of testosterone and cortisol may be used to detect potential disorders before observing clinical symptoms (e.g., overtraining, anxiety, and depression) [38]. Our results indicated an effect of time for both investigated hormones, while no between-activity differences were evident. These results seem in line with those reported in a systematic review and meta-analysis focusing on hormonal responses of HIIT sessions, which highlighted an increase of both hormones following HIIT sessions (testosterone in the range 0–30 min and cortisol 0–60 min) [38]. When considering 3 × 3 basketball activities, no previous study has assessed the effect of playing official or recreational 3 × 3 basketball matches on salivary cortisol and testosterone levels. However, when considering the cortisol levels, it should be noted that our results are similar to those obtained in semi-professional basketball players involved in playing 3 × 3 small-sided games played with different tactical tasks and a different training regime, [39] which indicated an increase of cortisol following any proposed small-sided games. Differently, the increase in salivary testosterone levels found in our study are not supported by previous literature in 3 × 3 basketball small-sided games, which showed either an increase, a decrease or no changes in salivary testosterone levels based on the combination of various training tasks and regimes [39]. It should be noted that inconsistent results were also found in a recent systematic review assessing the changes in salivary testosterone levels measured before and after official basketball matches [40], suggesting that further studies are necessary on the mechanisms related to testosterone levels following basketball activities. Overall, our study indicated that both activities increased the level of cortisol and testosterone in active young adults, which can be considered as important information about the anabolic and catabolic process elicited by recreational 3 × 3 basketball and HIIT.

### Perceptual and psychological responses

A higher RPE was found in HIIT compared to recreational 3 × 3 basketball. This result might be explained by the higher metabolic stress induced by the HIIT condition compared to the 3 × 3 basketball condition. However, this outcome is in contrast to the heart rate results, in which a higher %HR_max_ was observed in 3 × 3 basketball compared to HIIT activity. Furthermore, it should be noted that while the intensities elicited from both activities might be categorized as vigorous due to the high HR responses registered (> 77% HR_max_), interestingly, RPE responses do not match the vigorous definition since average values of 5 AU and 6 AU were reported, for recreational 3 × 3 basketball and HIIT, respectively, which are more representative of a moderate intensity [28]. Despite the higher cardiovascular intensities found in recreational 3 × 3 basketball, the low RPE values might be related to the nature of the activity, characterized by teammate interactions, which in turn can promote positive psychological responses, as previously observed in other recreational basketball activities [41].

The investigated players also reported much higher enjoyment during 3 × 3 basketball compared to the HIIT activity. A possible reason for this result might be that since all players were previously or currently recreational basketball players, they would enjoy participating in basketball-specific activities more than gym-based activities. The higher enjoyment elicited by playing recreationally 3 × 3 basketball together with the lower RPE values documented in this study could indicate that recreational 3 × 3 basketball might improve the long-term exercise adherence, which seems fundamental to be physically active.^10^

### Physical demand

Our findings showed that recreational 3 × 3 basketball elicited higher physical activity intensities measured via accelerometers compared to the HIIT condition with very large and moderate effect sizes reported for the percentage of time spent in the MVPA and vigorous activity, respectively. A possible explanation for this result could be the higher displacement likely attained during the recreational 3 × 3 basketball compared to the HIIT condition. Indeed, the adopted accelerometer, although considered one of the main tools to assess physical activity intensities in adults, [42] records accelerations while placed on participants’ hip and might not have been able to fully detect the physical demand of the activities performed during the HIIT, which were also characterized by gym-based static activities (e.g. push-ups and sit-ups). Therefore, this tool might not be the best solution for the analysis of the difference in the physical demand between the two studied conditions. It should be noted that its limitations were also indicated in assessing the physical demand in recreational handball, since the low physical intensities registered during recreational handball matches (27% and 10% of total match time in MVPA and vigorous activity, respectively) did not match the high physiological responses elicited (71% and 24% of total match time above 80% HR_max_ and 90% HR_max_, respectively) [22]. In contrast, our findings demonstrated that on average players spent 95.6% and 71.9% of the time in MVPA and vigorous activities, respectively, in the recreational 3 × 3 basketball condition, matching the physiological demand measured via heart rate monitors. The discrepancy in these results might be due to the fact that recreational 3 × 3 basketball matches are characterized by shorter activities and resting time compared to recreational handball matches. Indeed, during resting times, high heart rate responses might be recorded following high-intensity actions, while no or minimal physical demands could be recorded from the accelerometers, particularly during static actions. This might not be the case during recreational 3 × 3 basketball, in which players are continuously moving on the court with a short rest time, resulting in less possibility of a discrepancy between physiological and physical demand. Therefore, the adopted accelerometer might provide acceptable results for the analysis of recreational activities with a short rest time such as 3 × 3 basketball. However, this speculation should be further analysed with a future validation study.

### Limitations and future studies

The results of this study suggest valuable indications for health practitioners about the potential of recreational 3 × 3 basketball, which might produce similar or larger health benefits over time compared to HIIT. However, the results can be referred only to active young adults with previous basketball experience, and different results might be produced in other populations (youth or older adults), in people with different initial physical activity levels (sedentary people) or those with no or limited previous basketball experience. Therefore, future studies should expand our findings, investigating also youth and older adults, active and sedentary people and those with or without previous basketball experience. Furthermore, future investigations should employ an intervention design to assess whether recreational 3 × 3 basketball can elicit better or similar adherence and health benefits in comparison to HIIT.

## CONCLUSIONS

Our findings revealed that the analysis of the acute responses of recreational 3 × 3 basketball elicited higher %HR_max_, enjoyment, and physical activity intensities but lower BLa and RPE compared to HIIT in active young adults. Therefore, recreational 3 × 3 basketball might be considered as a potentially suitable activity able to increase the adherence and the health status in young adults.
